# Editorial: Neurosurgical treatment for neuro-ophthalmologic conditions: Intracranial pressure disorders

**DOI:** 10.3389/fopht.2022.1021667

**Published:** 2022-10-12

**Authors:** Marc J. Dinkin, Clare L. Fraser, John J. Chen, Susan P. Mollan

**Affiliations:** ^1^ Department of Ophthalmology, Weill Cornell Medical College, New York Presbyterian Hospital, New York, NY, United States; ^2^ Department of Neurology, Weill Cornell Medical College, New York Presbyterian Hospital, New York, NY, United States; ^3^ Save Sight Institute, The University of Sydney, Sydney, NSW, Australia; ^4^ Department of Ophthalmology, Mayo Clinic, Rochester, MN, United States; ^5^ Departments of Neurology, Mayo Clinic, Rochester, MN, United States; ^6^ Translational Brain Science, Institute of Metabolism and Systems Research, College of Medical and Dental Sciences, University of Birmingham, Birmingham, United Kingdom; ^7^ Birmingham Neuro-Ophthalmology, Queen Elizabeth Hospital, University Hospitals Birmingham National Health Service (NHS) Foundation Trust, Birmingham, United Kingdom

**Keywords:** idiopathic intracranial hypertension, venous stenosis, venous stenting, papilledema, fast track

A growing body of evidence has accumulated over the last two decades demonstrating dural venous sinus stenosis along the transverse sinus-sigmoid sinus junction in the majority of patients with idiopathic intracranial hypertension (IIH). This has led to a series of studies evaluating angioplasty and stenting of the stenotic segment in patients who are refractory to medical therapy. Two new studies published in Frontiers in Ophthalmology’s Collection, “Neurosurgical Treatment for Neuro-ophthalmologic Conditions: Intracranial Pressure Disorders,” add to this growing body of evidence, and are buttressed by careful quantitative neuro-ophthalmic assessment which have not been included in the majority of previous studies (1). In addition, this collection includes a manuscript describing a “fast track” strategy toward approaching patients with vision threatening disease from IIH that nicely summarizes when surgical intervention is required.

The first of two studies on venous sinus stenting in IIH was Reid et al., which offered several novel insights, including the observation that 18% of 226 consecutive IIH patients were defined as medically refractory and that 90% of these patients not only had stenosis but were shown to have a significant trans-stenotic gradient. Of those 32 stented patients, papilledema resolved following intervention in 96% with the average Humphrey visual field mean deviation improving from -4.42 dB to -1.45 dB. Optical coherence tomography (OCT) imaging demonstrated a reduction in the mean retinal nerve fiber layer (RNFL) thickness from 248 to 101 µm. Interestingly, of six patients who did not have papilledema on funduscopy, three demonstrated a reduction of RNFL on OCT following stenting, suggesting that occult disc edema was present at the time of stenting, highlighting the utility of OCT in quantifying subtle optic disc edema. In this study only 6.25% required a second procedure, which is lower than the average failure rate seen in the published literature (9-13%), but this may in part reflect the fact that five of the 32 patients had less than two months follow up at the time of analysis. No significant complications were reported although arachnoid granulation herniation through the stent mesh was observed in two cases and initially interpreted as thrombosis.

Oyemade et al. was the other in this collection that investigated venous sinus stenting, which included an impressively large cohort of 82 people with IIH treated with dural venous stenting from two centers over a 6-year period. Their assessment benefited from Frisén grading (2), which allowed them to demonstrate an average improvement in the grade from 1.76 to 0.39. This was associated with a reduction in the OCT RNFL from 186.34 to 96.86 µm. Interestingly, symptomatic improvement was moderate, with only 16.5% reporting complete resolution their headaches, likely reflective of the multiple contributors to headache in IIH patients (3). Of the 65 participants with pulsatile tinnitus at presentation and documentation of it at follow-up, pulsatile tinnitus completely resolved in 61.5%, which is less than the resolution rate among prior studies. The improvement in the visual field mean deviation (−4.87 to -3.79 dB) was not significant, owing to a relatively high proportion (33%) of patients with normal visual fields at baseline, but it is worthy of note that 42.3% of those with a mean deviation greater than -2 dB did show improvement after stenting. Finally, Oyemade et al. looked at the macular ganglion cell layer thicknesses (GCL) pre- and post-stenting, which has only rarely been reported on. Unlike RNFL, whose thinning can reflect either evolving optic atrophy or improvement in papilledema, GCL only thins in the setting of atrophy. The demonstration of stable GCL pre- and post-stenting in this large cohort may support the efficacy of stenting in preventing optic atrophy, which is the structural correlate of permanent visual field loss following papilledema, although the follow up period may have been too short to detect atrophy in some of the cases. Together these studies would support the use of dural venous sinus stenting in people with IIH and a stenosis as an alternative approach when medical therapy fails due to the documented clinical benefits and the low side effect profile. Ultimately, they pave the way for a large multi-center randomized control trial to provide class 1 evidence for the use of stenting for resolution of papilloedema and preservation of visual function in IIH.

At the present day, cerebrospinal fluid shunting remains the most common surgical treatment of IIH, and optic nerve sheath fenestration remains a viable option in many centers (4, 5). The importance of early identification of patients in need of escalation to a surgical intervention is increasingly being recognized. [Miri et al.] Likewise, there is no current consensus on the definition of medically refractory disease. In this collection, Reid et al defined medical refractory as intolerance or lack of effect at 1000mg acetazolamide daily in a divided dose. This level is lower than the maximum dose of 4g in a daily divided dose recommended by the IIH treatment trial (6). The literature also lacks robust data on which parameters define the need for surgical escalation to avoid permanent vision loss from IIH. To make a start on these clinically meaningful dilemmas, Miri et al. description of a “fast track” strategy for IIH patients for early diagnosis and early determination for the need of surgical intervention is a welcome addition to the literature. They propose neuro-ophthalmic assessment including papilledema grade, OCT imaging and formal visual fields, followed by radiological assessment to include venography (MRI/MRV or CT/CTV depending on the institution), and lumbar puncture as the initial steps in the protocol. Then, surgical intervention should be performed if warranted, and if not available, temporization with a lumbar drain or daily lumbar puncture can be performed. While such an algorithm may seem familiar to the practicing neuro-ophthalmologist, recognition of these steps in the emergency room setting may be understandingly variable. It is logical then that implementation of a formal fast track strategy, which has worked well in other diseases (7, 8), concentrates those specialists with the appropriate skills to move patients through investigations and towards management in a timely fashion. This has the clear potential to reduce emergency room visits, reduce the length of hospital stays and the number of return visits related to complications which are common in the current surgical treatments of IIH (9). Overall, fast track strategies have the ability to improve patient outcomes. We look forward to prospective trials assessing such a strategy to provide evidence for patient benefit and cost-effectiveness in IIH.

## Author contributions

MD made substantial contributions to the conception or design of the work, the acquisition, analysis, or interpretation of data for the work, drafting the work and revising it critically for important intellectual content, providing approval for publication of the content. CF made substantial contributions to the conception or design of the work, the acquisition, analysis, or interpretation of data for the work, drafting the work and revising it critically for important intellectual content, providing approval for publication of the content. JC made substantial contributions to the conception or design of the work, the acquisition, analysis, or interpretation of data for the work, drafting the work and revising it critically for important intellectual content, providing approval for publication of the content. SM made substantial contributions to the conception or design of the work, the acquisition, analysis, or interpretation of data for the work, drafting the work and revising it critically for important intellectual content, providing approval for publication of the content. All authors agree to be accountable for all aspects of the work in ensuring that questions related to the accuracy or integrity of any part of the work are appropriately investigated and resolved.

## Conflict of interest

MD reports a role as primary investigator for the River Trial (Serenity Medical) which evaluates venous stenting for medically refractory idiopathic intracranial hypertension. JC reports advisory board and consulting fees from UCB, Roche, and Horizon; all of which are not relevant to the topic of the submitted work. CF reports advisory board and consulting fees from Invex Therapeutics, and speaker fees from Roche. SM reports advisory board and consulting fees from Invex. Therapeutics and speaker fees from Heidelberg engineering. Other conflicts include: Chugai-Roche Ltd; Gensight; Janssen; Allergan;Santen; Roche; Neurodiem; all outside conditions of raised intracranial pressure which is the topic of the submitted work.

## Publisher’s note

All claims expressed in this article are solely those of the authors and do not necessarily represent those of their affiliated organizations, or those of the publisher, the editors and the reviewers. Any product that may be evaluated in this article, or claim that may be made by its manufacturer, is not guaranteed or endorsed by the publisher.
